# Prognostic significance of CTCs and CSCs of tumor drainage vein blood in Dukes' stage B and C colorectal cancer patients

**DOI:** 10.3892/or.2012.1649

**Published:** 2012-01-19

**Authors:** RYU SHIMADA, HISAE IINUMA, TAKUYA AKAHANE, ATSUSHI HORIUCHI, TOSHIAKI WATANABE

**Affiliations:** Department of Surgery, Teikyo University School of Medicine, Tokyo 173-0003, Japan

**Keywords:** colorectal cancer, circulating tumor cells, cancer stem cell, CD133, CEA, CK20, CK19, tumor drainage vein blood

## Abstract

The clinical significance of circulating tumor cells (CTCs) including cancer stem cells (CSCs) (CTC/CSC) in the tumor drainage vein blood of patients with colorectal cancer (CRC) is unclear. In this study, we investigated the prognostic value of CTC/CSC that express carcinoembryonic antigen (CEA) cytokeratin 19 (CK19), CK20 and/or CD133 (CEA/CK/CD133) mRNA in the tumor drainage blood of CRC patients with Dukes' stage B and C. We examined tumor drainage blood from 197 patients with Dukes' stage B and C CRC. CTCs that expressed CEA, CK19, CK20 and CD133 mRNA were detected using the quantitative real-time reverse transcription-polymerase chain reaction (RT-PCR) assay. Each mRNA level was normalized with GAPDH mRNA levels. In the relationship between the expression of CEA/CK/CD133 in the tumor drainage blood and clinicopathological factors, a significant correlation was observed between CEA/CK/CD133 expression and Dukes' stage (p<0.041). In CRC patients with Dukes' stage B and C, disease-free (DFS) and overall survival (OS) of patients with CEA/CK/CD133 positive in the tumor drainage blood were significantly worse than that of marker gene negative patients. In contrast, in patients with Dukes' stage A, no significant differences were shown between these groups. By Cox progression analysis, it was shown that CEA/CK/CD133 mRNA in tumor drainage blood was an independent prognostic factor for DFS and OS in patients with Dukes' stage B and C. These results suggest that detecting CEA/CK/CD133 mRNA in tumor drainage blood by the real-time RT-PCR method would have a prognostic value in CRC patients with Dukes' stage B and C.

## Introduction

Colorectal cancer (CRC) is one of the leading causes of worldwide cancer-associated morbidity and mortality (1). CRC is staged according to the extent of primary organ involvement and metastatic spread to lymph nodes or distant organs. The 5-year survival rates of CRC patients with Dukes' stage B and C who underwent surgery were 75–80% and 65–70%, respectively (2). Despite surgical resection being highly effective for localized disease, a significant proportion of these patients develop recurrence. Of particular concern is the fact that it is not possible to accurately differentiate between good and poor prognosis of Dukes' stage B. Effective biomarkers for the detection of Dukes' stage B patients who are at high risk are needed since the role of adjuvant chemotherapy in these patients remains controversial (3–7).

The utility of circulating tumor cells (CTCs) as biomarkers for predicting the clinical outcome of patients with various cancers has been shown by many studies (8–11). The detection of CTC in blood may not only provide the mechanism for the early metastatic spreading of isolated cancer cells, but it can also potentially indicate substantial predictive and prognostic information on CRC patients (9,12–15). In the detection of aggressive CTCs in blood, the possibility of a cancer stem-like cell (CSCs) marker is currently attracting attention (16). CSCs have been defined as a unique subpopulation in tumors that possess the ability to initiate tumor growth and sustain tumor self-renewal (17,18). Accumulating evidence shows that CSCs are associated with metastasis, resistance to chemotherapy and radiotherapy, and recurrence. It is known that CSC markers are frequently over-expressed in the CTC of patients with metastatic breast cancer, and most CTCs have CSC phenotypes that are not proliferating and resistant to chemotherapy (11,19). These properties suggest that the founder cells of metastases may arise from the CTC population. Recently, we demonstrated that detection of CTC, including CSC (CTC/CSC) in PB, is a useful tool for determining high risk for recurrence and poor prognosis in patients with Dukes' stage B and C CRC (16).

Peripheral blood (PB) and tumor drainage vein blood were used to obtain a CTC sample (9). As compared to the utility of CTCs in PB, the property of CTCs in tumor drainage vein blood is still unclear. It is known that the detection rate of CTCs in tumor drainage vein blood is higher than that of PB (9). Therefore, the detection of CTC/CSC in tumor drainage vein blood may show a high sensitivity for selection of the high risk patients for recurrence in Dukes' B and C patients. However, little is known about the clinical significance of CTC/CSC in the tumor drainage vein blood of these patients.

In this study, we aimed to clarify the usefulness of CTC/CSC in the tumor drainage vein blood as a prognostic biomarker in CRC patients with Dukes' stage B and C. To detect the CTC/CSC, we utilized the real-time RT-PCR method using multiple marker genes consisting of CEA, CK19 and CK20 mRNA for the general CRC-associated marker, and CD133 mRNA for the CSC marker.

## Patients and methods

### Patients

A total of 197 CRC patients (107 male and 90 female) with Dukes' stage B and C who underwent surgery at Teikyo University Hospital between 2000 and 2004 were enrolled in this study. The observation period ranged from 22 to 60 months, and the median follow-up period was 37 months. Study protocol conformed to the guidelines of the ethics committee of each university. Written informed consent was obtained from all patients. Their ages ranged from 27–82 years, with a mean age of 68 years. The stages of the tumors were determined according to the Dukes classification system. As a follow-up, all patients were re-evaluated at 3-month intervals during the first year, and then at 18 months, 24 months and yearly thereafter. Each evaluation consisted of a pertinent medical history, physical examination and repetition of imaging studies, including CT scans of the abdomen.

### Blood sampling and cDNA preparation

As controls, portal system blood samples collected from 20 patients with benign diseases were prepared. In CRC patients, blood samples were obtained from the mesenteric vein draining the tumor immediately after laparotomy. These samples were collected in PAXgene tubes, stored at −80˚C, and transferred to Teikyo University for real-time RT-PCR assay. Total RNA of the blood samples in PAXgene tubes was extracted using a PAXgene blood RNA Kit (Qiagen K.K. GmbH, Germany). Extracted total RNA was reverse transcribed into cDNA using oligo-p(dT)^12–18^ primers according to the manufacturer's protocol (Invitrogen Corp., CA, USA).

### Quantitative real-time RT-PCR

The primer and probe sequences of CEA, CK19, CK20, CD133 and glyceraldehyde-3-phosphate-dehydrogenase (GAPDH) have been previously described (16). GAPDH was utilized as an internal control. Real-time quantitative RT-PCR of these transcripts was performed with the LightCycler instrument (Roche Diagnostics, Mannheim, Germany) as previously described. The expression levels of CEA CK19, CK20 and CD133 were normalized by GAPDH, and the ratio of CEA, CK19, CK20 or CD133 copies to the GAPDH copies was calculated.

### Statistical analysis

The correlation between the presence of CEA, CK19, CK20 and CD133 mRNA in the blood and the various clinical parameters was evaluated using the chi-squared test. Disease-free survival (DFS) and overall survival (OS) were analyzed using the Kaplan-Meier method, and the differences were examined using the log-rank test. Univariate and multivariate analysis were performed using Cox regression analysis. P-values <0.05 were considered statistically significant.

## Results

### Cut-offs of markers and expression of CEA/CK/CD133 mRNA

The CEA, CK19, CK20 and CD133 mRNA levels were normalized with GAPDH mRNA levels in the portal system blood samples from 20 patients with benign diseases prepared for the control of tumor drainage blood to determine the cut-off levels. Based on the range of CEA/GAPDH, CK19/GAPDH, CK20/GAPDH and CD133/GAPDH, we determined the cut-off value by the 95% confidence intervals (mean plus 1.96 standard deviation) of the control groups. In the portal system blood samples, cut-off ratios were 5.7×10^−6^ in CEA/GAPDH and 9.5×10^−5^ in CK19/GAPDH, 2.9×10^−5^ in CK20/GAPDH and 4.7×10^−4^ in CD133/GAPDH. The positive rates, sensitivity and specificity of various combinations of genetic markers were examined (data not shown). As previous reported, CEA^+^, CK19^+^, CK20^+^, and/or CD133^+^ (CEA/CK/CD133) showed the highest positivity, sensitivity and specificity in the multimarker groups (16). Therefore, we selected the CEA/CK/CD133 group as representative of PCR positivity and used it for the following prognostic analysis.

### Relationship between CEA/CK/CD133 mRNA and clinicopathological factors

The relationship between the expression of CEA/CK/CD133 in the tumor drainage blood, and the clinicopathological factors was examined. A significant correlation was observed between CEA/CK/CD133 expression and the Dukes' stage (Table I). These results suggest that the presence of CTC in the tumor drainage blood correlated to the tumor progression.

### Kaplan-Meier OS and DFS curve analysis

**Figure 1:**
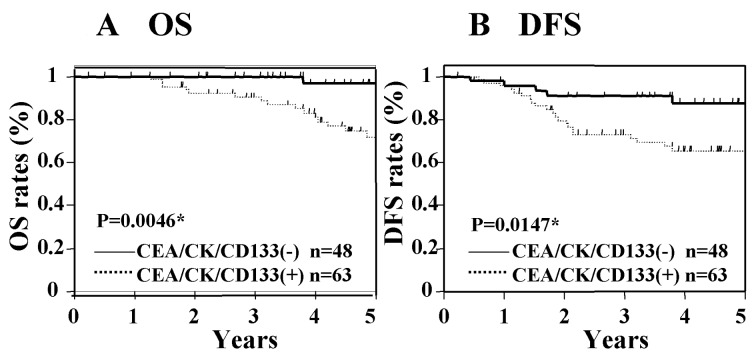
Kaplan-Meier survival curves for OS and DFS and status of CEA/CK/CD133 positivity in patients with Dukes' stage B cancer. Significant differences were shown between the CEA/CK/CD133 positive (+) and negative group (−). ^*^P-value<0.05.

**Figure 2:**
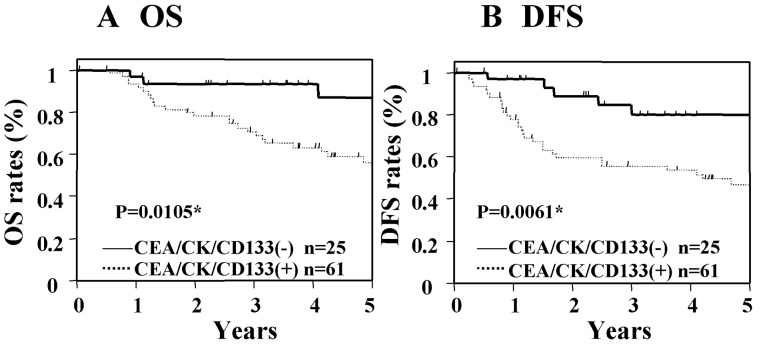
Kaplan-Meier survival curves for OS and DFS and status of CEA/CK/CD133 positivity in patients with Dukes' stage C cancer. Significant differences were shown between the CEA/CK/CD133 positive (+) and negative group (−). ^*^P-value<0.05.

The Kaplan-Meier survival curves show the OS and DFS rates according to the CEA/CK/CD133 gene expression status in CRC patients with Dukes' stage B and C. In this analysis, the average follow-up time for OS was 36.1±20.7 months and that of DFS was 38.8±16.2 months. Fig. 1 shows the OS and DFS in Dukes' stage B cancer according to the PCR status. The OS and DFS of those patients with CEA/CK/CD133 positivity were significantly worse than those who were negative for these markers (Fig. 1). In patients with Dukes' stage C cancer, the OS and DFS of the group with CEA/CK/CD133 positivity were significantly worse than those patients who were negative for these markers (Fig. 2). These results suggest that the expression of CEA/CK/CD133 in tumor drainage blood is associated with poor prognosis in patients with Dukes' stage B and C cancer.

**Figure 3:**
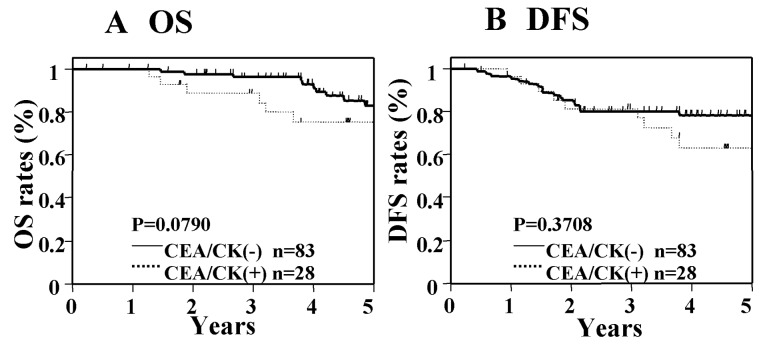
Kaplan-Meier survival curves for OS and DFS and status of CEA/CK positivity in patients with Dukes' stage B cancer. Significant differences were not seen between the CEA/CK positive (+) and negative group (−).

**Figure 4:**
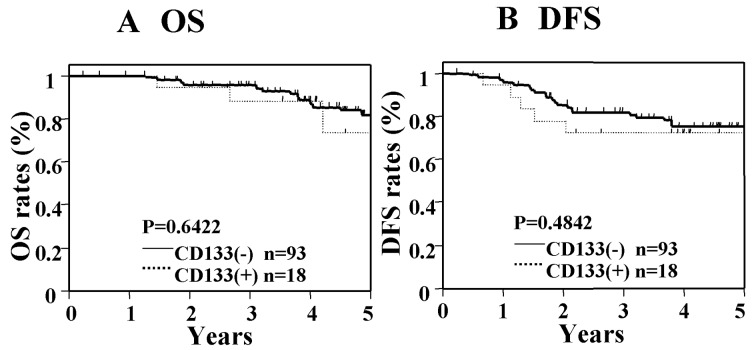
Kaplan-Meier survival curves for OS and DFS and status of CD133 positivity in patients with Dukes' stage B cancer. Significant differences were not seen between the CD133 positive (+) and negative group (−).

**Figure 5:**
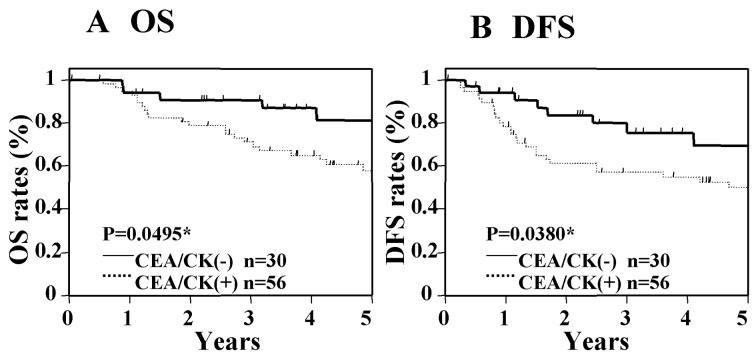
Kaplan-Meier survival curves for OS and DFS and status of CEA/CK positivity in patients with Dukes' stage C cancer. Significant differences were shown between the CEA/CK positive (+) and negative group (−). ^*^P-value<0.05.

**Figure 6:**
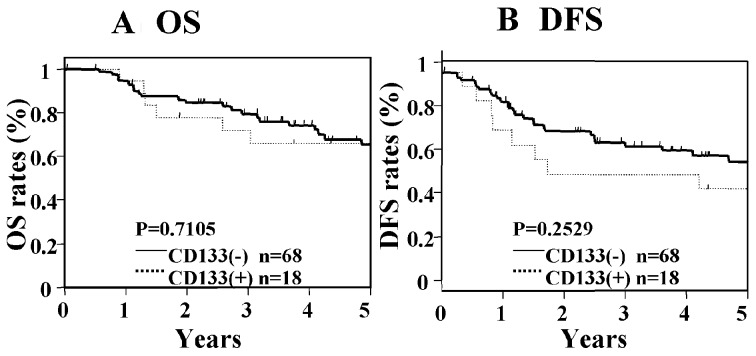
Kaplan-Meier survival curves for OS and DFS and status of CD133 positivity in patients with Dukes' stage C cancer. Significant differences were not seen between the CD133 positive (+) and negative group (−).

Next, we analyzed the OS and DFS in the general CTCs markers (CEA, CK19, and/or CK20: CEA/CK), and in the CD133 single marker. In patients with Dukes' stage B cancer, significant differences in OS and DFS were not seen between those who were positive for CEA/CK and those who were negative for CEA/CK (Fig. 3), nor were significant differences found between patients who were positive for CD133 as compared with those who were negative for CD133 (Fig. 4). In contrast, in patients with Dukes' stage C cancer, significant differences were seen in OS and DFS between those who were positive for CEA/CK and those who were negative for CEA/CK (Fig. 5). However, in the CD133 single-marker analysis, significant differences in OS and DFS were not found between patients who were positive for CD133 and those who were negative for CD133 (Fig. 6). These results suggest that the addition of CD133 to general CTCs markers is important for the determination of patients at high risk of recurrence and poor prognosis in Dukes' stage B cancer.

### Cox univariate and multivariate analysis of prognostic factors

Table II shows the results of univariate and multivariate Cox analysis of various factors for OS and DFS in patients with Dukes' stage B cancer. In univariate analysis of these patients, venous invasion and the CEA/CK/CD133 showed significance for OS and DFS. Then multivariate analyses were evaluated in factors which showed significance in univariate analyses. In these analyses, only CEA/CK/CD133 showed significance for OS and DFS. Table III show the results of univariate and multivariate Cox analysis of various factors for OS and DFS in patients with Dukes' stage C cancer. In univariate analyses of patients with Dukes' stage C cancer, lymphatic invasion, venous invasion, serum CEA and CEA/CK/CD133 showed significance for OS, and venous invasion, serum CEA, serum CA19-9 and CEA/CK/CD133 showed significance for DFS. In multivariate analyses, venous invasion and CEA/CK/CD133 showed significances for OS and DFS. These results suggest that CEA/CK/CD133 is an independent significant prognostic factor in patients with Dukes' stage B and C cancer.

## Discussion

Our study demonstrates that the detection of CEA/CK/CD133 mRNA in tumor drainage vein blood samples has prognostic significance in patients with Dukes' stage B and C CRC.

Evidence is rapidly accumulating that cancers are composed of heterogeneous populations of cancer cells. This suggests that CTCs may contain CSCs which are more aggressive and have invasive and metastatic capacity. We hypothesize that founder cells at the metastatic site may be CSCs disseminated from the primary tumor to a distant metastatic site. This hypothesis is supported by the similarities between the properties of CTCs and CSCs (19,20). It has been reported that CSCs or tumor-initiating cell markers are frequently overexpressd in the CTCs of patients with metastatic breast cancer, and most of them have CSC phenotypes that are do not proliferate and resistant to chemotherapy (19). Based on these reports, we selected multimarkers which included the general CTCs and the CSCs marker of CRC. Regarding the general marker of CRCs, several studies support the usefulness of CEA, CK19 and CK20 (9,10,15,21). In contrast, CSCs markers for CRC patients are still controversial (17). Previously, several interesting studies have demonstrated that CD133-positive cells of CRC have high tumorigenic ability in nude mice (22–24). Thereafter, it was reported that CD133-negative cells are capable of initiating CRC growth. The expression of EpCAM, CD166 and CD44 in CRC is also associated with aggressive tumor phenotypes (25). Thus far, several markers such as CD133, CD44, CD166, CD24, CD29, leucine-rich repeat-containing G-protein-coupled receptor 5 (Lgr5), and aldehyde dehydrogenase 1 (ALDH1) have been reported (24). Although CD133 may have limitation for CSCs, we elected the CD133, which is a key marker for CSCs. In this study, we used five genetic markers consisting of CEA, CK19, and CK20 for the general CTC markers, and CD133 for the CSC.

Next, we evaluated the prognostic value of CTC/CSC in the tumor drainage vein blood of patients with Dukes' stage B and C. Using general CTCs markers such as CEA and CK, many studies have reported a significant correlation between the presence of CTCs of PB and survival (10,21,26). Previously, we demonstrated that CEA/CK20 in the tumor drainage vein blood was an independent prognostic marker for OS and DFS when using Kaplan-Meier and Cox multivariate analysis (9). In contrast, no studies have examined the prognostic value of CTCs of tumor drainage vein blood in each Dukes' stage. In the present study, we have demonstrated that the detection of CEA/CK/CD133 in tumor drainage vein blood samples has prognostic value in patients with Dukes' stage B and C. To the best of our knowledge, our study is the first to demonstrate the prognostic significance of CTC/CSC in the tumor drainage vein blood of these patients.

Furthermore, to clarify the clinical value of CD133 addition in Dukes' stage B and C, we re-analyzed the prognostic significance of the general CTC marker (CEA/CK) group and the CD133 group separately using the Kaplan-Meier survival curve analysis. Our results demonstrated that CEA/CK/CD133, but not CEA/CK, is prognostic factor in patients with Dukes' stage B. In contrast, not only CEA/CK/CD133 but also CEA/CK showed significance in patients with Dukes' stage C. These results were similar to ones we obtained earlier when we examined PB samples (16). To date, the determination of patients with Dukes' stage B who are at high risk for recurrence is difficult, and efforts to find a tool to select them are ongoing. In this study, we used the real-time RT-PCR method, and it was difficult to examine whether individual cells express all markers or not. We speculate that CTC/CSC, which includes the CD133-positive cells, may enable identification of a certain subgroup that has aggressive cancer stem-like cell properties and that this may increase their prognostic value in Dukes' stage B and C cancer. As for the reason why the CD133 single marker did not show any prognostic value in the Dukes' B and C patients, we speculate that it may be due to the potential limitation of CD133 as CSC marker.

In this study, we established that CEA/CK/CD133 mRNA detection of tumor drainage vein blood is a useful tool for the determination of high risk patients with Dukes' stage B and C who are in need of postoperative adjuvant therapy.

## Figures and Tables

**Table I:** Relationship between clinicopathological factors and PCR positivity rates.

Variables	No. of patients (n=197)	PCR positive cases (n=124)	Positive rate (%)	p-value
Age (years)	68±11			
Sex				
Male	107	63	58.88	0.198
Female	90	61	67.78	
Tumor size (cm)				
<5	109	72	66.06	0.314
≥5	88	52	59.09	
Histological type				
Well differentiated	138	92	66.67	0.098
Poorly differentiated	59	32	54.24	
Lymphatic invasion				
Negative	130	83	63.85	0.715
Positive	67	41	61.19	
Venous invasion				
Negative	84	50	59.52	0.391
Positive	113	74	65.49	
Dukes' stage				
B	111	63	56.76	0.041^a^
C	86	61	70.93	

a P<0.05.

**Table II:** Univariate and multivariate analysis of prognostic factors for OS and DFS in Dukes' stage B patients.

	Univariate analysis			Multivariate analysis		
		
Variables	Regression coefficient	Hazard ratio (95% CI)	P-value	Regression coefficient	Hazard ratio (95% CI)	P-value
OS						
Tumor size	0.349	1.417 (0.521–3.858)	0.488		(−)	
Lymphatic invasion	0.674	1.962 (0.665–5.303)	0.209		(−)	
Venous invasion	1.109	3.030 (1.054–10.846)	0.039^a^	1.017	2.765 (0.961–9.905)	0.060
Histological type	−0.235	0.791 (0.181–2.455)	0.708		(−)	
Serum CEA	0.744	2.105 (0.768–5.777)	0.145		(−)	
Serum CA19-9	0.588	1.800 (0.394–6.235)	0.409		(−)	
CEA/CK/CD133	2.347	10.456 (2.119–189.993)	0.001^a^	2.284	9.819 (1.987–177.568)	0.002^a^
DFS						
Tumor size	0.053	1.055 (0.479–2.286)	0.892		(−)	
Lymphatic invasion	0.570	1.768 (0.775–3.844)	0.169		(−)	
Venous invasion	0.840	2.316 (1.040–5.645)	0.040^a^	0.619	1.857 (0.824–4.569)	0.139
Histological type	0.376	1.456 (0.597–3.242)	0.389		(−)	
Serum CEA	0.798	2.222 (0.823–4.901)	0.064		(−)	
Serum CA19-9	0.240	1.271 (0.360–3.546)	0.680		(−)	
CEA/CK/CD133	1.150	3.158 (1.286–9.462)	0.011^a^	1.282	3.602 (1.454–10.863)	0.005^a^

a P<0.05.

**Table III:** Univariate and multivariate analysis of prognostic factors for OS and DFS in Dukes' stage C patients.

	Univariate analysis			Multivariate analysis		
		
Variables	Regression coefficient	Hazard ratio (95% CI)	P-value	Regression coefficient	Hazard ratio (95% CI)	P-value
OS						
Tumor size	−0.273	0.761 (0.326–1.654)	0.498		(−)	
Lymphatic invasion	0.810	2.248 (1.048–5.004)	0.038^a^	0.647	1.910 (0.856–4.373)	0.114
Venous invasion	1.113	3.043 (1.243–9.110)	0.013^a^	1.156	3.178 (1.177–11.084)	0.021^a^
Histological type	0.401	1.493 (0.655–3.229)	0.328		(−)	
Serum CEA	0.838	2.313 (1.006–5.958)	0.048^a^	0.560	1.751 (0.755–4.543)	0.198
Serum CA19-9	0.721	2.056 (0.900–4.613)	0.086		(−)	
CEA/CK/CD133	1.439	4.215 (1.473–17.737)	0.005^a^	1.497	4.468 (1.537–18.948)	0.004^a^
DFS						
Tumor size	0.316	1.372 (0.692–2.696)	0.360		(−)	
Lymphatic invasion	0.271	1.311 (0.660–2.579)	0.434		(−)	
Venous invasion	1.263	3.535 (1.562–9.476)	0.002^a^	1.172	3.228 (1.383–8.834)	0.006^a^
Histological type	0.644	1.903 (0.934–3.777)	0.075		(−)	
Serum CEA	0.713	2.039 (1.008–4.373)	0.047^a^	0.555	1.742 (0.762–4.164)	0.190
Serum CA19-9	0.833	2.300 (1.133–4.622)	0.022^a^	0.291	1.337 (0.611–2.958)	0.466
CEA/CK/CD133	1.246	3.475 (1.466–10.222)	0.003^a^	1.253	3.502 (1.423–10.542)	0.005^a^

a P<0.05.

## Abbreviations

CRC: colorectal cancer
CTC: circulating tumor cells
CSC: cancer stem cells
RT-PCR: reverse transcription-polymerase chain reaction
CEA: carcinoembryonic antigen
CK20: cytokeratin 20
CK19: cytokeratin 19
GAPDH: glyceraldehyde-3-phosphate-dehydrogenase
